# The national portfolio for postgraduate family medicine training in South Africa: a descriptive study of acceptability, educational impact, and usefulness for assessment

**DOI:** 10.1186/1472-6920-13-101

**Published:** 2013-07-25

**Authors:** Louis Jenkins, Bob Mash, Anselme Derese

**Affiliations:** 1 Division of Family Medicine and Primary Care, Western Cape Department of Health, George Training Complex, University of Stellenbosch, George, South Africa; 2 Division of Family Medicine and Primary Care, University of Stellenbosch, Tygerberg, South Africa; 3 Centre for Education Development, Faculty of Medicine and Health Sciences, Ghent University, Ghent, Belgium

## Abstract

**Background:**

Since 2007 a portfolio of learning has become a requirement for assessment of postgraduate family medicine training by the Colleges of Medicine of South Africa. A uniform portfolio of learning has been developed and content validity established among the eight postgraduate programmes. The aim of this study was to investigate the portfolio’s acceptability, educational impact, and perceived usefulness for assessment of competence.

**Methods:**

Two structured questionnaires of 35 closed and open-ended questions were delivered to 53 family physician supervisors and 48 registrars who had used the portfolio. Categorical and nominal/ordinal data were analysed using simple descriptive statistics. The open-ended questions were analysed with ATLAS.ti software.

**Results:**

Half of registrars did not find the portfolio clear, practical or feasible. Workshops on portfolio use, learning, and supervision were supported, and brief dedicated time daily for reflection and writing. Most supervisors felt the portfolio reflected an accurate picture of learning, but just over half of registrars agreed. While the portfolio helped with reflection on learning, participants were less convinced about how it helped them plan further learning. Supervisors graded most rotations, suggesting understanding the summative aspect, while only 61% of registrars reflected on rotations, suggesting the formative aspects are not yet optimally utilised. Poor feedback, the need for protected academic time, and pressure of service delivery impacting negatively on learning.

**Conclusion:**

This first introduction of a national portfolio for postgraduate training in family medicine in South Africa faces challenges similar to those in other countries. Acceptability of the portfolio relates to a clear purpose and guide, flexible format with tools available in the workplace, and appreciating the changing educational environment from university-based to national assessments. The role of the supervisor in direct observations of the registrar and dedicated educational meetings, giving feedback and support, cannot be overemphasized.

## Background

Worldwide educational thinking and assessment of health professionals have moved towards a focus on competence in real world situations [1-4]. Public accountability has created a focus on outcomes, with an emphasis on abilities of the professional, de-emphasizing time-based training and promoting greater learner-centeredness [5]. Competencies in context, or entrustable professional activities, are an integration of the competencies that allow a doctor to perform the expected professional activities within a speciality [6,7]. Learning and being assessed in the workplace is underpinned by adult learning theory, which is essentially experiential, with Kolb’s learning cycle describing how a learner develops by observing experience, reflecting on that experience, planning the application of this learning, and implementing these plans such that a new experience is created [8-10]. Assumed in this process is supervisor observation of trainees with opportunities for feedback to improve performance. This has been very inadequate, with first year trainees in internal medicine not being observed more than once by a faculty member in a patient encounter involving history taking and a physical examination [11]. Among 1057 counselling sessions involving primary care physicians and surgeons, only 9% of encounters met basic criteria for effective informed decision making [12]. Other studies have shown that physicians fail to elicit over half of patient complaints and that many of the public’s complaints about physicians relate to communication problems [13].

Workplace-based assessment (WPBA) of clinical competence, directly observing trainees at work, has been recognised as a powerful means of changing learner behaviour, with evidence existing for its reliability and validity [14-17]. The shift in the focus of assessment has relevance for the design of high stakes assessment and for formative assessment [18]. Factors enhancing or impeding the efficacy of WPBA include the provision of feedback to the learner and the involvement of faculty in providing training to assessors [14]. Tips for improved observations include creating a culture that values direct observations, having role models, faculty development, feedback, action planning, observing multiple times, and embedding observations within usual patient care [19]. Other facilitators and barriers to implementing WPBA include translating the national curriculum to the local context, designing the curriculum with the needs of the primary users in mind, aligning the benefits directly with the outcomes of health care processes, such as patient safety, and have clear goals, time frames, and monitoring of results in place [3]. WPBA of postgraduate doctors in training is further influenced by the clinical context, patient complexity, passive versus proactive learner attitudes, supervisor involvement and evaluator versus coach attitude, informal versus formal learning processes, working versus learning agenda, and work-orientated versus training-orientated institutional culture [20-22].

We are still growing in our understanding of how to effectively evaluate clinical competence [23]. We need to stay open to conceptualizations of practice and identity formation, move beyond simply teaching supervisors how to use assessment tools and attempt to understand cognitive barriers, appreciating the value of feedback as a means of engaging trainees in assessment and professional development [23]. How professionals think in the workplace has been described as reflection-in-action and reflection-on-action, as part of an epistemology of practice [24]. A constructivist, social-psychological perspective has been proposed for the “messy” clinical realities in the “swampy lowland” of everyday patient care [24,25]. Learning as a process of controlling, changing or shaping behaviour was influenced by the field of humanistic psychology, which focused on personal development, self-initiated learning, evaluation by the learner, and personal meaning [26-28]. Learning has become not a task to be engaged with or a separate activity, but a way of being in the world, in contrast with critical theory, which is more concerned with the outcomes of learning, namely social change [29-31]. This tension between individual learning and society’s expectations is seen in clinical training programmes, where the learner (registrar) focusses on personal growth and meaningful learning, while health service managers and the public are interested in better health outcomes and service delivery.

From this new understanding of how learning takes place in the workplace, a growing body of evidence supports the role of a learning portfolio in both formative and summative assessments of competency in postgraduate training [32]. Many factors have been identified that influence the successful introduction of portfolios [33]. The purpose must be clear and aligned with the design of its content and structure. For example if it is more formative then it may be designed as a reflective journal, but if it is more summative then it may be designed as a collection of learning events. The changing learning environment, from lecturing to coaching and self-direction, in which the portfolio is used, plays a big role in its success [33]. The portfolio per se does not add any value to learning and assessment – it is only useful or valid to the extent that learners engage with it [34]. Experience with a learning portfolio in the clinical workplace suggest that trainees maintained a low view of its educational value, with barriers of a heavy workload, uncertain usability, and not getting feedback through their portfolios, while positive trainee attitude significantly correlated with greater perceived educational benefits [35]. With supervisors continuing to rely on feedback from clinical colleagues rather than portfolio evidence to monitor trainee development, trainees may battle to experience educational gains and disengage with the portfolio [35]. If feedback, particularly textual multisource feedback, is implemented well, there is evidence that it leads to a perceived positive effect on practice [36,37].

In South Africa (SA), learning portfolios have been used in postgraduate teaching in palliative medicine and undergraduate internal medicine education [38,39]. The submission of a portfolio of learning has now become a requirement for assessment of training by the Colleges of Medicine of South Africa (CMSA). In order to sit the Fellowship of the College of Family Physicians of South Africa, or FCFP(SA) examination, which is the national exit examination for postgraduate family medicine training, an acceptable portfolio, graded annually by each university, over 3 years, is required. A uniform national portfolio of learning has been developed, content validity has been established among the eight family medicine training programmes, and implemented with registrars in all eight departments of family medicine [40,41]. Workshops on reflective learning, portfolio completion, and supervision have been held at all the medical schools. The portfolio is not seen as the only or ultimate assessment method for training, but has become an increasing valuable tool assisting with the evaluation of clinical competence.

The setting of this study was the eight medical schools in six of the nine provinces in South Africa, each having a division or department of family medicine, with a 4-year postgraduate training programme that leads to a Master of Medicine (MMed) degree. This involves registrars working and learning in training complexes that include district and regional hospitals as well as primary care health centres and clinics. In the clinical setting registrars are supervised by family physicians as well as other specialists at the regional hospitals. Each training complex is linked to an academic programme run by one of the university medical schools. Ten clinical domains are covered, including mental health, general adult medicine, child health, women’s health, HIV/AIDS and infectious diseases, ear/nose/throat/eyes/skin, general surgery, emergencies, anaesthetics, and orthopaedics. Important principles and competencies are also addressed, including consultation and communication skills, ethics, professionalism and human rights, evidence-based practice, family-orientated care, personal and human growth and development, community-orientated care, chronic care, health promotion and disease prevention, management and administration, teaching, education and research [42].

The national portfolio consists of a lever arch file with dividers between the ten sections, with some electronic evidence or tools, made up of a the following items: An introduction to the portfolio, explaining the formative and summative purpose of the portfolio; a summary of the national learning outcomes for the discipline to be met in the portfolio [43]; personal learning plans for every clinical rotation, reflections on these rotations, with supervisor feedback and a summative assessment by the supervisor for every rotation; a record of various individual and group educational meetings with supervisors or peers, to include at least 24 hours per year [44]; at least ten direct observations by various supervisors, with feedback and summative assessments, with tools provided in the form of mini-CEX, observation of consultations or direct observation of procedural skills (DOPS) or a teaching event; various assignments with a summative grade that are required by the CMSA or the local university programme, including on ethics, community care, family care, teaching, communication, palliative care, and some elective assignments; a logbook with 216 nationally agreed skills that must be completed over four years [45], with guidance on the number and level of competency of procedures; and finally an assessment summary that collates individual summative assessment grades in the portfolio into a grade out of 100, with the program manager adding a global mark, particularly assessing the quality of reflections in the portfolio [46]. The contents of the family medicine portfolio is as follows:

1. Introduction to your portfolio

2. Learning outcomes

3. Learning plans, reflections on rotations and supervisor’s assessments

4. Educational meetings with the supervisor

5. Observations of the registrar by the supervisor

6. Written assignments

7. Logbook of clinical skills

8. Emergency medicine certificate(s)

9. Others courses, workshops, conferences

10.  End of year assessment

The aim of this study was to establish national registrar and supervisor portfolio engagement, especially whether users found the portfolio acceptable and practical to complete, useful for learning, and useful for summative assessment purposes.

## Methods

### Study design

The study was a descriptive survey of registrars and their supervisors who had used the portfolio during the previous year. The study was carried out in compliance with the Helsinki Declaration and approved by the Health Research Ethics Committee of the University of Stellenbosch, with Ethics reference number N09/10/258.

### Study population

The study participants were selected from across South Africa according to the following categories of expertise:

• Programme managers of the eight MMed programmes

• Family physicians responsible for supervision and training of family medicine registrars

• Family medicine registrars who had used the new national portfolio

The 104 registrars and 96 supervisors from the 8 postgraduate programmes in SA who were using the national portfolio were all invited to participate. This was done by personally contacting every university’s programme manager, explaining the study, and requesting the names and contact details of all supervisors and registrars who were using the portfolio. These were all invited by e-mail to participate.

### Data collection

Two structured questionnaires were developed, one for the family physician supervisors and one for the registrars. Construction of the questionnaires was guided by the literature [33,34,47-50]. The key sections in the questionnaires included: Participant demographics, understanding the purpose and reasons for completing the portfolio, whether or not the portfolio guide was read, extent of input into the portfolio, learning activities observed and feedback given, entry of written assignments, the clarity, feasibility, size and reasonability of the portfolio, how to improve the use of the portfolio, learning environment issues relating to work-training balance and secure academic time, educational meetings and the role of supervisors, and how to assess the portfolio. The question format was statements to be scored on a five point Likert scale, ranging from “strongly disagree” to “strongly agree”, or offering distinct categories (e.g. 1–2 weekly, 4 weekly, >8 weekly). After every block of questions, room was left open for comments. The questions were delivered using the internet-based software SurveyMonkey®, after piloting and revision.

### Data analysis

Data was analysed with the help of the Centre for Statistical Consultation at Stellenbosch University. Categorical and nominal/ordinal data were analysed using simple descriptive statistics. The answers “strongly agree” and “agree” were added up to one score “agree”. Pearson Chi-square was used to determine statistical significant differences between variables, defined as significant for a p-value < 0.05.

The responses to the open-ended questions were collated into a word document and analysed with ATLAS.ti version 6.2.27 software.

## Results

A total of 48 registrars and 53 supervisors responded (response rate of 50.5%), distributed across seven of the eight universities in the country. Of the 53 supervisors, 60% were male and 40% female; and of the 48 registrars, 71% were male and 29% female. The mean age of the supervisors was 47.2 years (SD 11.50), and the mean age of the registrars was 35.0 years (SD 5.15). Eight registrars (19.0%) were in their first year of academic training, 18 (43.0%) in their second year, 12 (28.6%) in their third year, and 4 (9.5%) in their fourth year of training.

### How practical and acceptable is the portfolio?

Most supervisors felt that the portfolio was practical and feasible to complete, while only half of the registrars agreed with this (Table 1).

**Table 1:** Supervisors’ (n=23) and registrars’ (n=26) views of the portfolio

	**Supervisors agree (%)**	**Registrars agree (%)**	**p-value**
I read the guide and portfolio together	23 (100.0)	20 (76.9)	0.01
Instructions in portfolio were clear	20 (86.9)	14 (53.8)	0.01
Portfolio was practical and feasible to complete	20 (86.9)	13 (50.0)	0.006
What was required in portfolio was reasonable	19 (82.6)	18 (69.2)	0.28
Portfolio reflects accurate picture of learning	16 (69.5)	14 (53.8)	0.26

**Figure 1:**
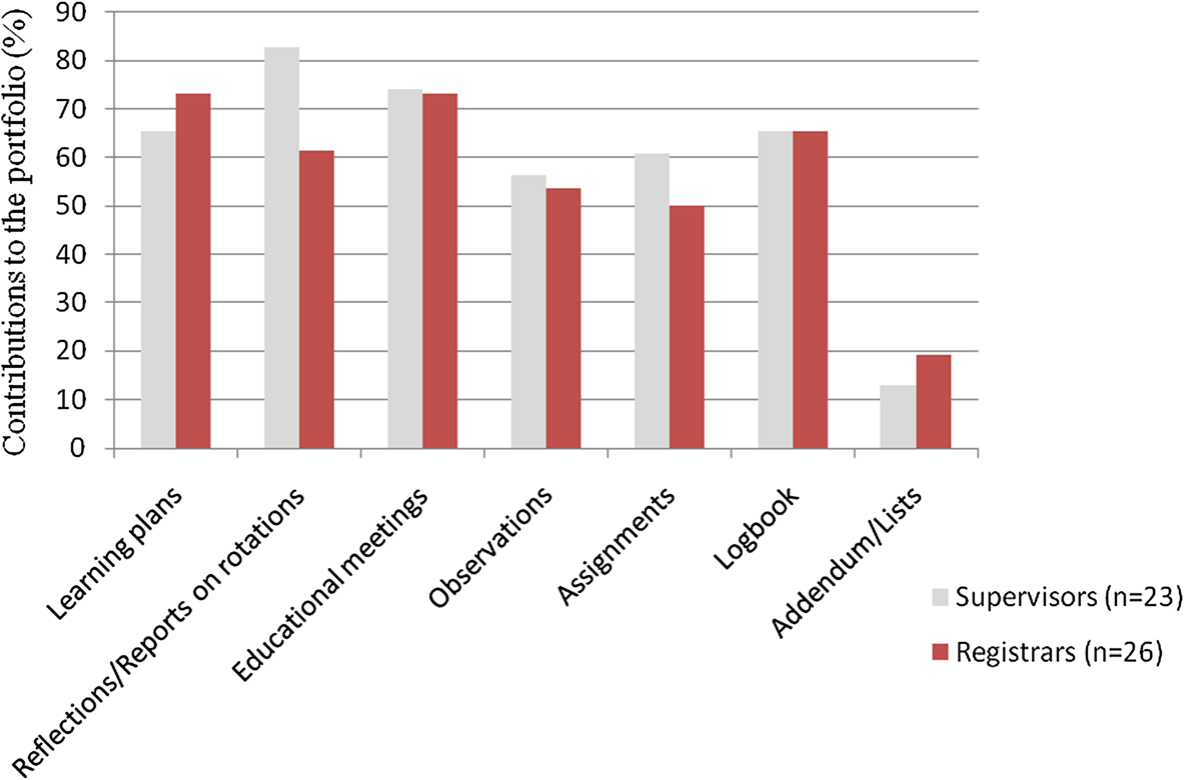
Contributions to the registrar portfolio by supervisors and registrars.

Overall engagement with the portfolio was lower than expected, particularly with the learning plans, logbook, written assignments and direct observations (Figure 1). In terms of reflections and reports on clinical rotations, 19 (82.6%) supervisors contributed a report and a grade, and only 16 (61.5%) registrars recorded their reflections on these rotations. Engagement with the new portfolio may have increased over time, as one registrar commented:

“*Initially I was quite reluctant to use the portfolio as it just seemed like another thing to do in an already full rotation*. *However*, *the more I have used it*, *the more I have appreciated its worth*.”

The commonest observation was of the consultation (n=33, 67.3%), followed by registrar’s teaching activities (n=28, 57.1%), and clinical procedures (n=25, 51%). Direct observation was used by all the supervisors, while only 4 (17.4%) supervisors and 5 (19.2%) registrars used video and 1 (4.3%) supervisor used audiotape for indirect observation. The assignments that registrars most frequently completed were on clinical competence (71.4%), evidence-based medicine (65.3%) and ethical reasoning (57.1%); followed by family orientated primary care (49.9%) and community orientated primary care (26.5%). The frequency with which registrars included these various types of assignments in their portfolio ranged from 12.2% to 51.0%.

Both groups agreed that the pressure of service delivery made training and portfolio completion difficult, as captured in the following quote:

“*We are already overwhelmed by the workload we face every day and putting that responsibility on our shoulders again will mean bending to breaking point*.”

Only 25 (51.0%) of registrars and supervisors combined thought that the portfolio helped to ensure clinical training took place during service delivery and 39 (79.6%) thought that the pressure of service delivery made completion of the portfolio difficult.

#### Understanding the portfolio

While most of the supervisors felt that the instructions in the portfolio were clear, only about half of the registrars agreed with this (Table 1). All the supervisors, but only 76.9% of the registrars read the portfolio with the portfolio guide (Table 1). Fifteen (65.2%) supervisors said their registrars understood how to use the portfolio, while 14 (53.8%) registrars thought their family physician supervisors understood how to use the portfolio. While 12 (54.5%) supervisors thought that family physicians and other specialist supervisors understood how to use the portfolio, 22 (91.7%) registrars thought that non-family physician supervisors did not adequately understand how to use the portfolio. Only 21 (42.8%) supervisors and registrars had attended a workshop on how to use the portfolio in the current year, of which 90.0% found the workshop useful. Responses to the open questions frequently asked for more guidance in using the portfolio.

#### Time and format

Considering secure academic time for portfolio completion, 37 (75.5%) supervisors and registrars agreed that 1–3 hours per week was sufficient. Some felt it was labour intensive and expected too much from the registrar and supervisor. Forty one (83.6%) were happy with the A4 size. Registrars suggested that the portfolio would be more practical if examples of what was required and a checklist of the essential items were included, with more regular deadlines to check progress, and a better layout of the contents. There was support for having the various tools and forms that could be included made more available, preferably electronically (e.g. internet). There was agreement that reflections should be captured as they happened. Registrars were more in favour of a brief daily dedicated time for this, using written notes in a journal or laptop, rather than an audio-recorder. Registrars liked the idea of a daily time for writing and reflection, including weekly dedicated time to update their portfolios. More regular interaction with the supervisor and other registrars specifically on the portfolio were supported, as well as motivating other specialists to be more supportive of their portfolios.

#### Ownership

Table 2 shows the respondents’ views on who takes ownership of the portfolio and what motivates them to complete it. Overall the registrars found it more useful in enabling reflection on learning than the supervisors’ believed was the case (p=0.03). Supervisors were more inclined to believe it was completed because it was a university regulation. While all agreed that registrars had to take responsibility for completing the portfolio, the registrars wanted the supervisors to take more responsibility:

“*With regards to the learning events*, *ultimately it is my responsibility to enter it*, *but if the supervising family physician is not often available or approachable*, *it is difficult to negotiate*.”

**Table 2:** Ownership and motivation to use the portfolio

	**Supervisors agree n=23 (%)**	**Registrars agree n=26 (%)**	**p-value**
They only complete the portfolio because it is a requirement of the university	21 (91.3)	20 (76.9)	0.17
They complete the portfolio because it helps them reflect on what they have learnt	10 (43.5)	19 (73.1)	0.03
They complete the portfolio because it makes their learning needs clearer	12 (52.2)	15 (57.7)	0.70
They usually only work on the portfolio when there is a university deadline	17 (73.9)	14 (53.8)	0.15
The supervisor should take responsibility for ensuring that the registrar’s learning events are observed and captured in the portfolio	7 (30.4)	16 (61.5)	0.03
The registrar should take responsibility for ensuring that his/her learning events are observed and captured in the portfolio	22 (95.7)	21 (80.8)	0.11

### What is the perceived educational impact of the portfolio?

Respondents were asked about the perceived educational impact in terms of the four stages of Kolb’s learning cycle [10]. There was strongest support for the portfolio’s ability to help registrars observe (43, 87.8%) and reflect on their experience in a way that made learning explicit (42, 85.7%). There was slightly less support for its ability to plan future changes in clinical practice (36, 73.5%) and actually implement them (33, 67.3%). Interestingly, while 16 (69.6%) supervisors thought that registrars were reluctant to record mistakes or negative experiences in their portfolio, only 11 (42.3%) registrars agreed. The majority, 35 (71.4%), reported that the portfolio also helped to integrate learning from theory, i.e. journal articles or textbooks, with clinical practice.

#### Educational meetings

Table 3 combines the responses of registrars and supervisors to give a picture of how frequently and what types of educational meetings took place. Educational meetings were required for completion of several sections of the portfolio. It is encouraging that 86% reported 1–2 weekly meetings with someone who could facilitate their learning. Meetings with a family physician took place less often and with their specific supervisor the least of all. There was no significant difference in the frequency of educational meetings between working in a regional or district hospital. Thirty four (69.4%) supervisors and registrars agreed that meetings were planned, while the same number reported that ad hoc meetings also occurred. While most meetings were taking place as expected, of note is that the setting of a learning agenda never took place in 14.3% of participants, and direct observations of clinical skills infrequently in 30.6%, and never in 12.3% of participants. The discussion of personal problems featured quite prominently, happening daily to monthly for 59.2% of participants.

**Table 3:** Frequency and types of educational meetings [N=49]

	**1-2 weekly n (%)**	**4 weekly n (%)**	**≥ 8 weekly n (%)**
An educational meeting between the registrar and someone who can facilitate their learning happens	42 (85.7)	4 (8.2)	3 (6.1)
A face-to-face meeting between the registrar and supervisor happens on average	31 (63.2)	8 (16.3)	10 (20.5)
If the registrar(s) work in a regional hospital how often do they meet with a family physician?	34 (69.4)	9 (18.3)	6 (12.2)
If the registrars work in the district, how often on average do they meet with a family physician?	37 (75.5)	6 (12.2)	6 (12.2)
	**Daily to weekly**	**2-4 weekly**	**≥ 8-weekly**
Setting a learning agenda	4 (8.2)	14 (28.6)	24 (48.9)
Intermittent evaluation of progress	2 (4.1)	22 (45)	23 (46.9)
Observation or demonstration of communication/procedural skills	13 (26.5)	15 (30.6)	15 (30.6)
Discussion of patients/cases	22 (44.9)	19 (38.7)	5 (10.2)
Critical reflection or appraisal of scientific literature	13 (26.5)	24 (48.9)	10 (20.4)
Academic programme/research issues	10 (20.4)	28 (57.1)	6 (12.3)
Personal problems	10 (20.4)	19 (38.8)	8 (16.3)

#### Feedback

Feedback to the registrars regarding their learning and progress was perceived by both groups, but especially the registrars (88.5% vs. 52.2%, p=0.005), to be very inadequate. As expected most feedback was received from family physicians and other specialists. The registrars reported less feedback from family physicians and other specialists and more from other registrars, medical officers, patients, or nurses, than the supervisors thought. Both groups reported little feedback from managers. Communication happened most often in one-to-one meetings (22, 84.6%) and group meetings (22, 84.6%), less via e-mail (16, 61.5%), and very seldom via the internet (7, 26.9%).

### How useful is the portfolio for assessment?

Table 4 shows the respondents’ views on how the portfolio should be assessed. In terms of summative assessment of the portfolio, 11 (47.8%) supervisors and 14 (53.8%) registrars said the portfolio should be graded at the end of rotations, while 9 (39.1%) supervisors and 7 (26.9%) registrars said it should be graded once a year, and 3 (13.0%) supervisors and 5 (19.2%) registrars said twice a year. All the respondents agreed that all the sections in the portfolio should be assessed, but the registrars felt less strongly about including the acquired number of entries for specific items, e.g. number of observations.

“*The number* (*ten*) *of observed consultations during the year is extremely difficult to achieve*. *Often one is working in specialized departments whose consultants do not value spending their time watching you consult with a patient*, *and the primary health care facilities are too busy to take time for those consultations*.”

**Table 4:** Agreement on sections that should contribute to the assessment of the portfolio

	**Supervisors n=23 (%)**	**Registrars n=26 (%)**	**P-value**
Achieving the goals in the learning plan	22 (95.6)	23 (88.4)	0.36
Achieving the required number of entries for specific items e.g. number of observations	20 (86.9)	15 (57.7)	0.02
Evidence of improvement during the year e.g. improvement in rating of skills	21 (91.3)	20 (76.9)	0.17
Evidence of critical reflection on one’s experience, growth and development e.g. reflections on rotations	23 (100.0)	23 (88.4)	0.09
Assessments by the supervisor e.g. at the end of rotations	23 (100.0)	24 (92.3)	0.17
Assessments given for specific items in the portfolio during the year e.g. assignments included in the portfolio	20 (86.9)	23 (88.4)	0.87
The logbook satisfactorily completed	22 (95.6)	22 (84.6)	0.20
The overall presentation and layout	17 (73.9)	19 (73.1)	0.95

There was some uncertainty with the logbook as a tool used for assessment, with comments such as this:

“*I find the logbook confusing to complete*. *I am unsure whether it is supposed to be a tool for directly observed procedures or just a general self*-*reporting tool*. *It also does not have enough space to keep adding as you learn and grow*.”

## Discussion

### How acceptable and feasible is the portfolio?

#### Guide and purpose

Almost a quarter of registrars had not read the guide to the portfolio and approximately half did not find the portfolio clear, practical or feasible. Supervisors had a more positive perspective, but non-family medicine supervisors were perceived to have little understanding of the portfolio requirements. The reasons for this may include that people had not studied the guide sufficiently, that the guide was not clear enough, or that people simply felt overwhelmed. It could also indicate a mismatch between their understanding of learning and the actual learning environment within which the portfolio was embedded. It is well described that a condition for success of portfolio learning is that it should be carefully embedded in an overall guidance system [34,51]. There needs to be clarity in the minds of the registrars and the supervisors about the goals (or purpose) of the portfolio [33]. The portfolio included a formative feedback component to the registrar, a summative graded component to the local training programme and achievement of an acceptable standard for entry to the national college examinations. The portfolio has been introduced in the context of changing from university-based examinations towards national college-based examinations, which may have complicated the acceptance of the portfolio [33]. As the college exam relieves the local training programmes of the need to conduct their own final summative evaluation, the portfolio may be given more weight and importance in the local setting. The usefulness of workshops on the use of the portfolio was evident and indicated a need for on-going engagement of registrars and supervisors on how to learn, reflect, supervise and assess in the workplace, and document this in the portfolio. Consequently, extensive workshops with all staff have been conducted on portfolio learning in all eight medical schools in the country. The portfolio guide is no longer a separate document, but has been incorporated as an introduction into the portfolio, with a short explanation around process, content and assessment for every section.

#### Time and format

There was strong agreement to have a brief dedicated time every day to reflect and write in their portfolios. It is important to capture reflections as they happen. The use of a journal or a diary, or an electronic tablet or laptop was strongly supported. This, together with the support for more regular meetings between the registrars and supervisors to discuss their portfolios, with protected weekly academic time, is imperative if formative feedback, and by implication, also summative assessment in the workplace, is to happen in a meaningful and valid sense [51]. Both supervisors and registrars agreed that the current pressure of service delivery makes training and portfolio completion difficult. Ultimately an electronic portfolio will be ideal, but has its own challenges [52]. In the meantime, the structure of the paper-based portfolio has being revised to incorporate the suggestions from this study.

### Educational impact: How did the portfolio help the registrar to learn?

While most of the supervisors felt that the portfolio reflected an accurate picture of learning, just over half of the registrars agreed with this. In contrast, most registrars reported that they completed the portfolio because it helped them reflect on what they have learnt, while less than half of the supervisors thought this was the case. Comparing with the four stages of Kolb’s learning cycle the impact of the portfolio was perceived to gradually decrease from observing and reflecting on their clinical experience, and to clarify through abstract conceptualization what they have learnt from that experience, to planning improvement and implementing change in clinical practice [9,10]. The impact of the portfolio on planning improvement correlates with the observation that learning plans were recorded in just over 70% of cases.

#### Educational meetings

Educational meetings between registrars and supervisors provide the opportunity for a range of different types of learning conversations [44]. While most participants reported that this happened 1–2 weekly, which is encouraging, these learning conversations were only being captured in the portfolio in just over 70% of cases, making it difficult to know how much learning took place and how to assess it. The frequent discussion of personal problems is perhaps not unusual, and points to the importance of a clinical supervisor who is concerned for the growth of the whole person [53,54]. Working in a harsh environment with competing expectations and threat of burnout makes the support and understanding of the supervisor critical not only to registrar learning, but also to resilience in the situation [55,56]. This may be related to registrar drop out. It also relates to the kind of personal growth in terms of one’s whole life, purpose and meaning that is happening at this stage of the lifecycle as people also try to make sense of who they are and where they are going. The importance of this may be more than we think, and is probably more pronounced in more developing countries with high disease burdens and low staffing ratios.

#### Supervision and feedback

While all agreed that ownership of the portfolio rested with the registrars, the registrars wanted the supervisors to take more responsibility around supervision, educational meetings, and giving feedback. It is important that the portfolio of learning belongs to the registrar, and that the registrar has the full support of a supervisor [49]. A clinical supervisor knows the registrar personally and should be able to evaluate authenticity and depth of portfolio contributions [52]. The supervisor needs to support the registrar’s learning by helping to extract the maximum benefit from what occurs [57]. Feedback was perceived by both groups, but especially the registrars, to be very inadequate, with less feedback from family physicians and other specialists and more from another registrar, medical officer, patient, or a nurse, than the supervisors thought. These latter groups are under recognised for their contributions, and could be incorporated more formally into the portfolio [58]. A wider variety of useful learning conversations could be included beyond the usual individual meetings with a supervisor, such as brief “corridor conversations”, mutual mentoring (self-help pairs or small groups of registrars), telephone mentoring (particularly if supervisor and registrar are working in different sites), and group-based mentoring [49]. Supervisors need tremendous support and structure to be able to supervise as is expected from them [59]. Training in skills of listening and giving feedback, awareness of self and others, peer support, being recognized and rewarded, protected time, and assessment are all important [59]. As most supervisors are not employed by the university their ability to perform these training and teaching roles relies on collaboration with the Department of Health and a commitment from them to enable and support these roles [42].

### How useful was the portfolio for the assessment of the registrar?

#### Reflections and clinical rotations

While the supervisors graded most of the rotations, suggesting that they understood the summative aspect, the registrars reflected on their rotations in only 61% of cases, suggesting that the formative aspects of the portfolio are not yet utilised as expected. This is not surprising, considering the low levels of feedback reported, the need for more protected academic time indicated, and both groups indicating the pressure of service delivery. Previously identified factors for successful implementation of a portfolio include clearly communicated goals and procedures, integration with curriculum and assessment, flexible structure, support through mentoring, and combining formative and summative assessment [34]. Our results show that we are on a learning curve applicable to all these factors. We need to be comfortable with the ‘implementation dip’, which can take a few years, before portfolios become accepted, during which time listening to people’s doubts and adapting is necessary [52,60].

#### Observations

Both the supervisors and registrars agreed that all the sections in the portfolio should be assessed, but the registrars felt less strongly about including ten direct observations of their competencies. This may have been because of the difficulties in finding a suitable observer. We know that assessment of competence is highly dependent on context and content, and to achieve reliable assessment we need a large sample (typically 8–10) across the curriculum [61,62]. The portfolio has been developed to include observations of a number of different skills, observed by different supervisors, in different settings, to increase the reliability and validity of the assessment [62].

#### Assignments

Assignments, particularly those relating to clinical competence, evidence-based medicine and ethical issues were entered into the portfolio in 51.0% or less of cases. Typically, assignments are done as part of the academic programme and may not be fully integrated with clinical training and supervision. For more valid assessments, these assignments need to move from the “high, hard ground” closer to the “swampy lowlands” and to be engaged with more fully by the supervisor in the clinical context [24]. As validity relies on authenticity and integration of competencies in a complex environment, it is precisely this integration of theory, learning and practice that the portfolio aims to encourage and capture [62].

#### Recommendations

Moving towards service-based learning and workplace-based assessment relating to the outcomes for family medicine training in South Africa [43,63], this study provides some evidence that the portfolio can work, provided some conditions are met, particularly:

1. Having a clear purpose and guideline for the portfolio.

2. Having the portfolio tools available in a feasible and flexible format, to facilitate entries and assessments, slowly moving from paper-based to an electronic format.

3. Building ownership of the portfolio by the registrar.

4. Creating realistic expectations about the time investment involved, securing perhaps 20 minutes daily for portfolio entries, and weekly or 2-weekly 30–60 minutes for learning conversations and feedback with the supervisor.

5. Investing in competent trainers through recognition, training workshops, secure academic time, and feedback from registrars and training programmes.

6. Embedding the portfolio in everyday practice, with the registrar taking advantage of both planned and opportunistic learning.

7. Supporting the registrar in each part of the learning cycle.

8. Developing resilience through attention to personal growth and development.

9. Developing an assessment tool that includes all sections of the portfolio and which provides reliable feedback on the progress of the registrar and acceptability of the portfolio for entry to the national exam.

10. Maintaining a strong formative focus at the level of the local training programme.

11. Allowing time for the portfolio to find its place in the workplace and to be accepted by registrars and supervisors.

12. Future work should include exploring an electronic portfolio with access via mobile technology.

#### Limitations

The number of study participants was relatively small, with 50% non-responders. From telephonic and e-mail inquiries to supervisor and registrar non-responders, the main reason given was lack of time to complete the rather long online questionnaire. No specific pattern or bias could be detected in the non-responders. The national postgraduate portfolio of learning is a recent introduction in family medicine in South Africa. Only seven out of eight medical schools participated, which might have skewed the results. However the medical school that did not participate is similar to the other medical schools, and had just started to implement a learning portfolio. It is unlikely that their inclusion would have changed the results significantly. About equal numbers of registrars and supervisors contributed. The registrars and supervisors were not paired, mostly because supervisors and registrars tend to rotate, which means we could not directly compare the opinions of a registrar with those of his/her concurrent supervisor.

## Conclusion

The introduction of a national portfolio of learning for postgraduate training in family medicine in South Africa faces challenges similar to those reported from high income countries. The acceptability of the portfolio relates to a clear purpose and guide, a flexible format with tools available in the workplace, and appreciating the changing educational environment from university-based assessment to national College-based assessment into which the portfolio is being introduced. The role of an available supervisor in direct observations of the registrar and dedicated educational meetings, giving feedback and support, cannot be overemphasized. This is a particular challenge in low-resource countries like South Africa, and may well apply to similar countries. Summative and formative assessment via the portfolio is a realistic expectation.

## Competing interests

The authors declare that they have no competing interests.

## Authors’ contributions

All three authors contributed to the planning and design of the questionnaire. LJ collected the data and analysed the results. All three authors contributed to the writing of the article. All authors have read and approved the final manuscript.

## Pre-publication history

The pre-publication history for this paper can be accessed here:

http://www.biomedcentral.com/1472-6920/13/101/prepub
